# Barriers and enablers to local active travel during COVID-19: A case study of Streetspace interventions in two London boroughs

**DOI:** 10.12688/wellcomeopenres.19164.1

**Published:** 2023-04-18

**Authors:** Maria Lunetto, Oscar Castro, Chiara Gericke, Joanna Hale

**Affiliations:** 1 Centre for Behaviour Change, University College London, London, England, WC1E 6AE, UK; 2 Future Health Technologies, Singapore-ETH Centre, Campus for Research Excellence And Technological Enterprise, National University of Singapore University Town, 138602, Singapore

**Keywords:** active travel, infrastructure, walking and cycling, behaviour change, intervention, COVID‑19

## Abstract

**Background: **During the coronavirus disease 2019 (COVID-19) pandemic, UK local authorities increased emergency active travel interventions. This study aimed to understand what aspects of temporary Streetspace for London schemes represent barriers or enablers to walking and cycling for short local journeys.

**Methods: **Focusing on two Inner London boroughs, we conducted 21 semi-structured stakeholder interviews and sampled 885 public comments about Streetspace schemes. We triangulated the data in a thematic analysis to identify barriers and enablers, which were categorised using the Capability, Opportunity, Motivation, Behaviour (COM-B) model.

**Results: **Opportunity and motivation factors were reflected in the barriers (accessibility and integration of the schemes; controversy, dissatisfaction, and doubt) and enablers (new routes and spaces; sustainability and health beliefs) and mixed themes (changes to traffic and appeal of the area; feelings of safety). Capability was not reflected in the main themes.

**Conclusions: **Although aspects of Streetspace schemes were seen to enable active travel, our findings suggest that additional processes to address the acceptability, fairness, and unintended consequences of emergency interventions will be important to their long-term success for health and sustainability.

## Introduction

During the coronavirus disease 2019 (COVID-19) pandemic local authorities in the UK faced urgent pressures to help people maintain physical distancing on streets and public transport systems ( Corker *et al*., 2021; SYSTRA, 2021). Although national and local travel restrictions and stay-at-home orders greatly reduced travel during periods of lockdown, necessary short local journeys for essential shopping, exercise, and some outdoor socialising could continue ( Priddy, 2021). Many local authorities sought to introduce or increase measures to promote the use of active travel for these types of journeys, particularly in urban areas. An added motivation was that promoting active travel is a goal of many strategies at local, national, and international level to achieve more sustainable transport systems and reduce greenhouse gas emissions ( Brand *et al*., 2021; Hirst & Dempsey, 2020; HM Government, 2021; Petrokofsky & Davis, 2016; UNEP, 2017). This is a particularly high priority in cities, which face growing mobility demand as urban populations grow ( Cavoli, 2021; Wegener, 2013). Increasing the modal share of active travel (i.e. the proportion of journeys made by walking, cycling, and other non-motorised modes) is also expected to have co-benefits for cardiovascular fitness and mental health ( Amelung *et al*., 2019; Avila-Palencia *et al*., 2017; de Nazelle *et al*., 2011; Keall *et al*., 2018; Rissel, 2009) as well as reducing air, water, and noise pollution caused by vehicle use ( de Nazelle *et al*., 2011; Douglas *et al*., 2011; GEA, 2012).

Across the UK, local authorities implemented a variety of schemes under emergency legislation to give more space and priority to walking and cycling ( Gore *et al*., 2021). They included creating protected cycleways and widening pavements, typically through installing temporary physical barriers. Other types of schemes use modal filters designed to restrict vehicles and discourage driving for short local journeys. The most prominent examples are low traffic neighbourhoods (LTNs), which commonly use bollards, planters, or other street furniture accompanied by signs to restrict through traffic on certain streets, and school streets, in which streets are closed to all vehicles during school opening and closing times, sometimes using temporary barriers placed outside by school staff. These were methods of traffic management introduced before the pandemic, but in May 2020 the UK government announced £250m funding to encourage local authorities to accelerate and increase their roll-out ( Department for Transport, 2020). Under the name ‘Streetspace for London’, Transport for London administered funding for London boroughs to adopt and reinforce these measures ( Transport for London, 2020), representing the largest coordination of interventions to support active travel in the UK to date.

It is unclear to what extent these kinds of temporary active travel schemes could lead to a ‘moment of change’, with lasting shifts in travel behaviours and expected health and environment co-benefits ( Corker *et al*., 2021; Marsden & Docherty, 2021; Nikitas *et al*., 2021; Nurse & Dunning, 2020). This is uncertain for several reasons. First, it is hard to separate the effects of the interventions from the effects of lockdown, during which home-working and cycling increased while public transport and car use declined ( Corker *et al*., 2021; Department for Transport, 2022; Harrington & Hadjiconstantinou, 2022). Aldred & Goodman (2021) found that the introduction of emergency LTNs in London were associated with more walking, some (but less clear-cut) reduction in driving, as well as a more positive perception of the cycling environment. It is unclear whether these results generalise to other regions of the UK and will be sustained in the long term, although research pre-dating the pandemic suggests that both LTNs and segregated cycleways can be relatively cost-effective and straightforward ways measures to increase the modal share of active travel and benefit health ( Aldred, 2016; Goodman *et al*., 2020; Laverty *et al*., 2021).

Secondly, it is possible that the expected benefits of active travel schemes, such as a decline in road injuries ( Laverty *et al*., 2021), could be diminished by unintended consequences associated with their rapid roll-out or other aspects. For example, there is little evidence about how these interventions interact with social inequalities around transport availability and accessibility, including greater dependency on public transport among people of lower socio-economic status ( Finbom *et al*., 2021; Gutiérrez *et al*., 2021; Joelsson & Ekman Ladru, 2022; Nazroo *et al*., 2020). Concerns about unintended consequences for emergency service response times and crime rates were not supported by initial studies of LTNs introduced in London during the pandemic ( Goodman *et al*., 2021; Goodman & Aldred, 2021). However, disabled-led groups have raised accessibility concerns around physical features such as cluttered and uneven pavements and narrow cycling lanes, and have reported that LTNs negatively impact people’s independence ( Transport for All, 2021).

 Thirdly, it is difficult to disentangle the efficacy of the measures from their acceptability; many were met with considerable controversy and resistance from local residents ( McIntyre, 2021; Plimmer, 2021) and some have subsequently been reversed. A qualitative study of residents' experiences with LTNs in Greater Manchester highlighted heated debates about their fairness, the implementation process, and possible monitoring and evaluation processes, which echo media reports in other regions ( Larrington-Spencer *et al*., 2021; Prosser, 2021; Wolmar, 2020). Opponents of the active neighbourhoods were often not motorists, as one might assume, but included people who walk exclusively for their local journeys. Disagreements have also arisen over tension between environmentalism and disability rights ( Transport for All, 2021).

To help unravel this complex picture, we aim to investigate what aspects of the temporary schemes and their implementation could act as barriers or enablers to walking and cycling locally. Evaluating the measures in this way could improve our understanding on why they may or may not promote active travel as intended and inform the development or improvement of future interventions ( Larrington-Spencer *et al*., 2021; Michail *et al*., 2021). While many existing barriers and enablers to active travel are known from previous research, these could be expected to differ in the context of the pandemic and temporary transport interventions. For example, enablers such as the presence of segregated cycle lanes and the experience of less traffic and pollution ( Christmas *et al*., 2010; Hull & O’Holleran, 2014; Keall *et al*., 2018; Kerr *et al*., 2016) might be enhanced in this context. On the other hand, barriers such as poor health and fitness ( Bauman *et al*., 2008) or financial resources to purchase active travel equipment ( Ravensbergen *et al*., 2020) could be exacerbated due to the immediate and long-term effects of COVID-19 on work and health ( del Rio *et al*., 2020). Safety concerns about injuries and bike theft ( Christmas *et al*., 2010; Manaugh *et al*., 2017; Marquez *et al*., 2018) might also be affected by new infrastructure and perceptions about how this changes the nature of the local area. Importantly, demographic groups who are disproportionately negatively affected by the pandemic coincide with groups who experience more (or more pronounced) financial and social barriers to active travel, including women, people with disabilities, older people, people of lower socio-economic status, and people from ethnic minorities ( Burns *et al*., 2020; Goodman & Aldred, 2018; Marmot *et al*., 2020).

**Figure 1.**
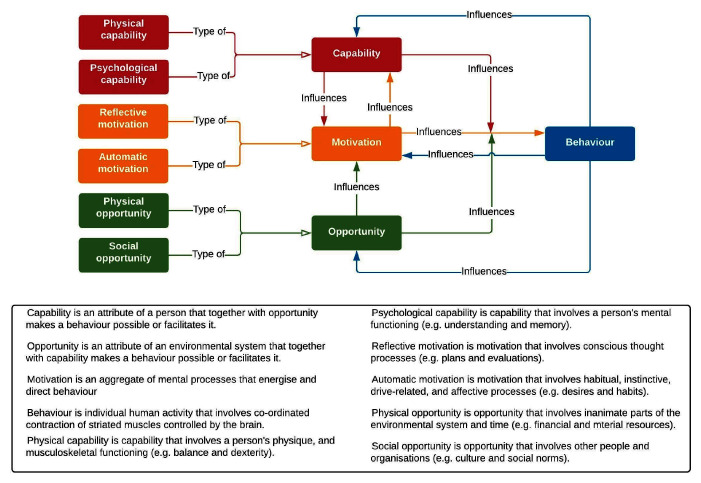
The COM-B Model of Behaviour. *Note*. This figure has been reproduced with permission from West, R., & Michie, S. (2020). A brief introduction to the COM-B Model of behaviour and the PRIME Theory of motivation. *Qeios*. https://doi.org/10.32388/WW04E6.2.

Behavioural science models offer a systematic way to investigate factors acting as barriers to (preventing, discouraging) or enablers to (supporting, encouraging) particular behaviours such as walking or cycling. One of the most simple, yet comprehensive and widely-used models is the Capability, Opportunity, Motivation, Behaviour (COM-B) model ( Michie *et al*., 2011) which proposes that these three conditions are necessary for any behaviour to occur and can be further sub-divided as shown in Figure 1 ( West & Michie, 2020). The COM-B model has been applied to understanding health and transport behaviour during the pandemic ( Burton *et al*., 2021; Corker *et al*., 2021) as well as environmentally significant behaviours ( Addo *et al*., 2018; Allison *et al*., 2021; Graça *et al*., 2019; Hale *et al*., 2022a; Kolodko *et al*., 2021). An advantage of using this model is that it forms the basis of the wider Behaviour Change Wheel framework and toolkit for designing and evaluating interventions ( Michie *et al*., 2011; Michie *et al*., 2014; West *et al*., 2019).

There is extensive literature on the barriers and enablers to active travel. Some of the most cited barriers to cycling are safety concerns while riding, and security concerns for storing bikes ( Aldred & Jungnickel, 2013; Manaugh *et al*., 2017; Pooley *et al*., 2013). Relative route comfort including distance, hilliness, perception of noise and air pollution, aesthetics, weather conditions, and darkness have been identified as key influences to active travel ( Heinen *et al*., 2011; Parkin *et al*., 2008; Pooley *et al*., 2013). There are a number of intraindividual factors that affect travel choices. These include health condition, lack of knowledge of road rules, skills and fitness, and time ( Bauman *et al*., 2008). Intentions, attitudes, and perceived behavioural control are also predictors of choosing alternative modes to cars ( Hoffmann *et al*., 2017). Clear trends also emerge in terms of demographic indicators. Groups that cycle less frequently include women, people with disabilities, older people, and people from ethnic minorities ( Goodman & Aldred, 2018). The latter, on average, feel less confident with their cycling skills and are more likely to see the cost of a bicycle as an obstacle compared to ethnic majorities ( Burns *et al*., 2020). For a review and synthesis of conceptual frameworks for active travel behaviour, see Götschi *et al*. (2017).

There are a wide range of types of interventions to increase active travel. Changing the built environment is the most common type of approach ( Kärmeniemi *et al*., 2018; Smith *et al*., 2017). Physically separating motorised traffic from cyclists and pedestrians (e.g., cycling lanes, widened pavements, traffic-free zones, and LTNs), installing zebra crossings, and providing safe parking spaces to minimise the risk of theft, vandalism, and weather damage have shown robust positive effects on active travel ( Aldred, 2016; Kärmeniemi *et al*., 2018; Petrunoff *et al*., 2017; Pooley *et al*., 2013; Pucher & Buehler, 2008; Ransdell *et al*., 2013; Smith *et al*., 2017). For reviews of walking and cycling interventions and environmental determinants, see Aldred (2019), Barnett *et al*. (2017), Foster *et al*. (2018), Fraser and Lock (2011), Larouche *et al*. (2018), Panter *et al*. (2019), and Smith *et al*. (2017). However, many active travel interventions have mixed effectiveness ( Aldred, 2019; Larouche *et al*., 2018; Panter *et al*., 2019) and there are evidence gaps around reasons for differences, since few interventions explicitly report the behavioural techniques or mechanisms expected to bring about change to walking and cycling behaviour ( Aldred, 2019; Bird *et al*., 2012).

In this study we aimed to use the COM-B model of behaviour to understand what aspects of temporary cycleways, widened pavements, LTNs, and school streets could act as barriers or enablers to walking and cycling for short local journeys. In particular, we focus on measures introduced as part of the Streetspace for London funding scheme, which could have the potential (if successful and maintained) to substantially change active travel behaviour in London, with major benefits to climate, health, and social fairness. The aim of this study was not to evaluate the success of the schemes in changing active travel rates, since the councils conducted an evaluation on this during the study period. The research question we seek to answer is: What are the barriers and enablers to walking and cycling for short local journeys in areas where Streetspace measures have been introduced? By better understanding the barriers and enablers presented by Streetspace for London measures, we aim to gain insights into whether and how these measures could be effectively scaled up from temporary, localised benefits efforts to long-term, city-wide improvements. Using the COM-B model in this way presents a novel approach, as typically it is used to inform intervention design, rather than analysing a behaviour in relation to an existing intervention. This study and its aim are part of a wider ongoing programme of research into Complex Urban Systems for Sustainability and Health (CUSSH; Belesova *et al*., 2018; Davies *et al*., 2021; Moore *et al*., 2021), which seeks to understand what types of interventions can deliver climate and health benefits for cities through participatory research with local stakeholders.

## Methods

### Design

This was a qualitative study focusing on two case study boroughs in Inner London. We triangulated from two complementary sources of data: (1) in-depth semi-structured interviews with invited stakeholders about their experiences of short local journeys and schemes in their area and (2) publicly available online comments from self-selecting members of the public about schemes in each borough. Our triangulation approach was done for two purposes: to help achieve greater *completeness* in answering our research question and to help look for *confirmation* between the two datasets ( Breitmayer *et al*., 1993; Fusch *et al*., 2018). The two datasets were expected to complement each other based on their diverging qualities. Online comments represent a large number of people’s views about a range of specific schemes, but have limited depth or elaboration, whereas interviews offered the opportunity to explore experiences in more detail with a targeted range of stakeholders. Given that this approach allows to capture very different views, triangulation was deemed a valuable approach. The methods are described in accordance with the Consolidated criteria for reporting qualitative research (COREQ) checklist for qualitative research ( Tong *et al*., 2007; Supplementary File 1, https://osf.io/pa9wv) and the 15-Point Checklist of Criteria for Good Thematic Analysis ( Braun & Clarke, 2006; Supplementary File 2, https://osf.io/56pye).

### Case study boroughs

The London boroughs of Hackney and Lambeth were selected as case studies because of existing links with the CUSSH project and their complementarity. They are broadly comparable in some respects such as population, size, location, and poverty rates, but differ in others. Notably Hackney has lower car ownership rates, a bigger proportion of average daily journeys is made by walking, cycling or public transport ( Barrett *et al*., 2020), and more Streetspace schemes were requested ( Mayor of London, 2020). Some key characteristics are reported in Table 1. Given the similarities and differences between the boroughs, the findings were expected to include a wide range of views which could be meaningfully integrated and aid generalisability.

**Table 1.** Case study borough characteristics.

Characteristic (Reference)	Lambeth	Hackney
Size (square miles) ( ‘List of London Boroughs’, 2022)	10.36	7.36
Location	South London	North-east London
Poverty Rate (2019/20) ( Trust For London, 2022)	26%	29%
Population (est. 2020) ( Office for National Statistics, 2020)	322,000	281,000
Car Ownership rate (2015–2018) ( Barrett *et al*., 2020)	41%	33%
Daily Journeys made by Walking, Cycling, and Public Transport ( Barrett *et al*., 2020)	77%	84%
Number of Streetspace schemes requested ( Mayor of London, 2020)	42	55
Amount of Streetspace funding (% of total funded) ( Mayor of London, 2020)	£1,013,700 (6.98%)	£1,452,000 (10.00%)

In each borough, four main types of Streetspace measure were introduced: temporary cycleways, widened pavements, LTNs, and school streets. Because these kinds of measures are intended to work together and different residents may encounter multiple or none of them on a given journey, we did not seek to analyse each type of intervention independently but instead explored them as an overall package.

Officers of each borough council were approached by the research team, provided feedback on the proposed research questions and methods, and took part in interviews. This research was not commissioned or endorsed by either council and was carried out independently by University College London.

### Ethical approval

The study was conducted in accordance with the Declaration of Helsinki, and ethical approval was obtained from the University College London Research Ethics Committee (January 2020, Reference: CEHP/2020/579) and the UCL Bartlett School of Environment, Energy and Resources (BSEER) Ethics Committee (January 2018). All interview participants gave written informed consent to take part. Informed consent was not sought for the analysis of online comments as these were published anonymously in the public domain.

### Interview participants

An initial list of potentially relevant stakeholder groups was identified by the researchers and reviewed by borough council officers for completeness. These included: (i) council officers; (ii) businesses; (iii) the health and social care sector; (iv) schools and families; and (v) community groups and residents. Local organisations were contacted by email or telephone to invite members to take part. Participants were also identified through referrals (snowballing). Participants were eligible to take part if they were familiar with at least some (though not necessarily all types of) Streetspace schemes in their borough, based on an information sheet illustrating types of schemes (Supplementary file 5). The types of schemes participants reported they were familiar with are summarised in Table 2. Priority was given to those also identifying with traditionally underrepresented groups in this type of research, which we identified in discussion with council officers as including people with disabilities, older people, faith groups, sexual and gender minorities, and ethnic minorities. Following APA style we use person-first language (i.e., ‘people with disabilities’), but recognise that other people and organisations prefer ‘disabled people’. Participants were offered compensation of a £10 voucher for their time, excluding council officers. A total of 21 participants took part ( Table 2). Five participants representing community groups and residents also identified themselves as belonging to underrepresented groups (people with disabilities, older people, and sexual and gender minorities). No participants we contacted through health and social care sector organisations were available to take part. None of the interview participants refused to participate or dropped out of the study after agreeing to take part.

**Table 2.** Participants by borough, stakeholder group, modes of travel used, and types of scheme encountered.

Stakeholder Group	Total	Hackney	Lambeth	Resident in borough	Works in borough	Cycles	Walks	Drives	School Streets	LTNs	Cycle Lanes	Widened Pave-ments
Council officersa	5	3	2	2	5	-	-	-	-	-	-	-
Businesses	3	0	3	2	3	2	3	2	1	3	3	2
Schools and families	3	2	1	3	3	2	3	1	3	3	3	2
Community groups and residents	10	4	6	10	4	6	10	4	10	9	10	6
Total	21	9	12	17	15	10	16	7	14	15	16	10

*Note.* Information on council officers’ modes of travel used and types of scheme encountered were not sought.

### Interview materials

An interview guide was developed using the COM-B model and the Theoretical Domains Framework (TDF) (Supplementary File 3, https://osf.io/j2y87; and Supplementary File 4, https://osf.io/mwj28). The TDF is an integrative, theory-based framework of behaviour change constructs, representing a more granular version of the COM-B model that can be used to systematically identify behavioural influences ( Cane *et al*., 2012). COM-B and TDF have been widely applied to inform interview guides and surveys in the health and sustainability domain ( Burton *et al*., 2021; Kolodko *et al*., 2021; Ojo *et al*., 2019; Zhang & Hale, 2022). The interview guide was pilot tested with two research assistants from UCL and updated in line with their feedback, before being used with study participants. To allow participants to engage in a natural flow of conversation, the TDF did not provide structural specification, but rather served to semantically inform the interview guide development ( McGowan *et al*., 2020). The interview guide followed the ‘funnelling’ technique, by which broader questions are asked first, followed by more detailed questions or prompts ( Morgan, 1997). At the start participants were asked about the Streetspace schemes they were familiar with in their borough. The following questions were asked about the schemes in general, rather than specific types. Although participants were guided to talk about Streetspace schemes, the discussion could also explore their experiences of local journeys more generally. An adapted version of the interview guide was produced for council officers to reflect their role in implementing the schemes and not necessarily encountering them in their personal journeys (Supplementary File 4, https://osf.io/mwj28).

### Interview procedure

Interviews were conducted between May and August 2021. Participants read a Participant Information Sheet about the study and gave informed consent before taking part. Before the interview took place, participants (excluding council officers) were sent an information sheet about Streetspace schemes in their borough, which was presented again by the researcher at the start of the interview (Supplementary File 5, https://osf.io/tjp5q). Interviews lasted approximately 45–60 min and were conducted via videoconferencing (Microsoft Teams and Zoom) by one of the researchers (ML, CG, or JH). Field notes were made after the interviews to capture immediate thoughts. The interviews were audio recorded, transcribed verbatim by a transcription service, and pseudonymised during the transcription process to remove identifiable information.

### Online comments

In February 2021 we sampled publicly available online comments about schemes introduced in Lambeth and Hackney from Commonplace, a widely used web platform used by local authorities beyond the case study boroughs to share information and collect feedback on new initiatives (including for statutory purposes). The comments covered periods of up to 6 months prior to sampling.

At the time of sampling the data, England was in its third national lockdown, schools were closed, strict rules prevented social gathering indoors or between more than two people outdoors, and vaccines were just becoming available. Through the Streetspace for London scheme, Hackney had introduced 33 school streets schemes, 2 temporary segregated cycle routes, and 15 low traffic neighbourhoods under experimental traffic orders and were collecting feedback on these at Hackney Council (2020). Lambeth had introduced 19 school streets schemes and 7 low traffic neighbourhoods under experimental traffic orders and were collecting feedback at Lambeth Council (2020). Lambeth had also introduced three road upgrades to cycle routes which were listed on the Commonplace platform but were not open for comments at the time we collected data.

Members of the public could visit the Commonplace webpages to leave anonymous feedback on individual schemes (e.g. specific LTNs) or types of schemes (e.g. school streets in general in Hackney). They would be prompted to answer survey questions administered by the council, which included a free-text question for general comments. All submitted feedback is displayed on Commonplace where others may read, agree, and share comments to social media.

For the purposes of answering our research question, and due to differences in the survey questions across different schemes, in the present study we sampled the free-text general comments and did not include any other types of response or information about the commenter. We extracted comments which were left on schemes at the above webpages up to February 12, 2021 (N = 15,065) and collated comments by type of scheme (school streets, low-traffic neighbourhoods, and cycling infrastructure changes). Note that there were not online comment data available for widened pavements, although interviews could touch upon this type of scheme.

### Analyses

**Figure 2.**
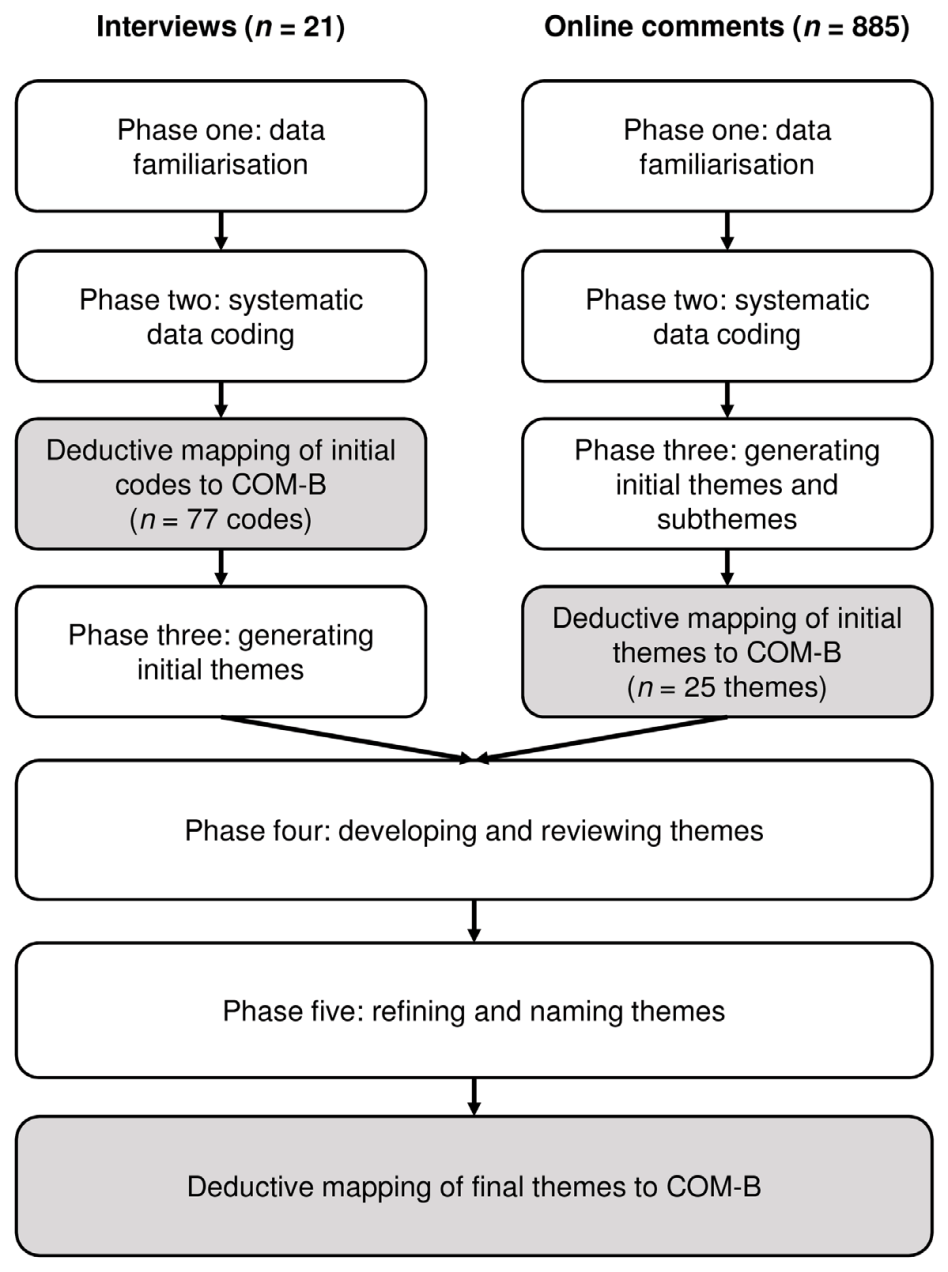
Overview of thematic analysis approach with deductive mapping to COM-B. *Note.* Flow diagram showing steps in our inductive thematic analysis approach (unshaded boxes) with deductive mapping to COM-B (grey shaded boxes). The final themes were triangulated from the interviews ( *n* = 21) and online comments ( *n* = 885).

To triangulate the data from the interview transcripts and the online comments, we used inductive thematic analysis, followed by deductive mapping of themes to the COM-B model of behaviour. COM-B was selected as one of the only models of behaviour which is comprehensive and linked to a wider framework for intervention design. It has many previous applications to health and transport behaviour. Other frameworks which have been used in comparable contexts and broadly consider similar factors are ISM and social practice theory ( Hargreaves, 2011; Scottish Government, 2013). We followed Braun & Clarke’s ( 2006; 2012; 2021) phases of thematic analysis. Phases 1–3 (data familiarisation, systematic data coding, generating initial themes) were carried out separately for the interview transcripts and online comments. In phases 4 and 5 (developing and reviewing themes; and refining, defining and naming themes) we combined the initial themes of the two datasets into one set of triangulated themes. Data analysis was performed using Nvivo 2020 software. Figure 2 summarises the overall steps in the analysis.

Epistemologically, our analyses were grounded in an essentialist/realism paradigm ( Bhaskar, 2013). We aimed to identify and describe barriers and enablers to the target behaviour using language relatively close to the surface or explicit meaning of the participants’ comments, rather than at the interpretative or latent level. Such approaches are considered useful for under-researched topics like the subject of this study ( Kim *et al*., 2017; Neergaard *et al*., 2009).

***Interview transcripts***

Steps in generating initial themes from the interview transcripts are summarised in Table 3.

**Table 3.** Steps in analysing the interview transcripts.

Phase of thematic analysis	Steps in analysis of interview transcripts
1. Data familiarisation	Three researchers (ML, CG, JH) were assigned transcripts to read and note recurring patterns or ideas (JH: council interviews, *n* = 5. CG: remaining Lambeth interviews, *n* = 10. ML: remaining Hackney interviews, *n* = 6).
2. Systematic data coding	Each researcher coded one-third of their assigned transcripts to generate initial codes. The researchers jointly developed a codebook including illustrative quotes and definitions. Each researcher applied the codebook to their assigned transcripts. Code labels and definitions were refined through regular communication between the researchers. Three researchers (ML, CG, JH) double-coded seven of 21 interview transcripts (rater pairings: JH, ML: council interviews, *n* = 1. CG, ML: remaining Lambeth interviews, *n* = 3. ML, CG: remaining Hackney interviews, *n* = 3). Subsequently, Cohen’s kappa and a percentage agreement score were calculated.
3. Generating initial themes	All researchers independently organised codes into tentative groupings. The researchers deductively mapped the initial themes to domains of the COM-B model and categorised them as barriers, enablers, or mixed. One researcher (ML) grouped codes into initial themes, which were reviewed by two researchers (JH, CG). Initial themes which did not directly address the research question but may be of interest were noted and retained.

***Online comments***

Steps in generating initial themes from the online comments are summarised in Table 4. To determine the final sample of online comments coded, we followed guidance on assessing thematic saturation in qualitative research ( Guest *et al*., 2020). According to this method, an initial number of comments (the ‘base size’) was pre-defined as N = 150 per type of scheme in each borough (school streets, low-traffic neighbourhoods, and cycling infrastructure changes). Further consecutive batches of N = 15 comments (the ‘run length’) were then analysed until no new codes were identified (‘new information threshold’). A final sample of 885 comments were analysed, available at https://osf.io/w9suy (Supplementary File 6). Of these, 375 were on school streets (180 Hackney; 195 Lambeth), 165 on cycling lanes (165 Hackney), and 345 on low traffic neighbourhoods (165 Hackney; 180 Lambeth). Note that there were not data on pavement widenings.

**Table 4.** Steps in analysing the online comments.

Phase of thematic analysis	Steps in analysis of online comments
1. Data familiarisation	Three researchers (OC, CG, JH) read a sub-sample of 420 comments and noted any recurring patterns or ideas.
2. Systematic data coding	Two researchers (OC, CG) independently generated initial codes during familiarisation. Three researchers (OC, CG, JH) discussed the initial codes as a group and produced a combined codebook. OC used the codebook to re-code the sub-sample of comments and code further comments until saturation was reached.
3. Generating initial themes	All researchers independently organised codes into tentative groupings. OC reviewed the tentative groupings and organised codes into initial themes and subthemes. All researchers discussed and revised the initial themes and subthemes. The researchers deductively mapped the initial themes and subthemes to domains of the COM-B model and categorised them as barriers, enablers, or mixed. Initial themes which did not directly address the research question but may be of interest were noted and retained.

***Development of triangulated themes***

Table 5 summarises the steps in combining the initial themes from both datasets into one overall set of triangulated themes.

**Table 5.** Steps in developing triangulated themes from interview transcripts and online comments.

Phase of thematic analysis	Steps in developing triangulated themes
1. Developing and reviewing themes	Three researchers (ML, CG, JH) reviewed all initial themes and subthemes. Through discussion all researchers agreed that initial subthemes generated from the online comments were described at a similar level of specificity to codes generated from the interviews and these could be organised into overall themes reflecting contributions from both datasets. One researcher (ML) organised *codes* from interviews and *initial subthemes* from online comments into overall themes, which were revied by two researchers (CG, JH).
2. Refining, defining and naming themes	Through iterative discussion and referring back to the data (both text fragments and coding frequencies), three researchers (ML, CG, JH) refined the themes and labels until they agreed each theme described a coherent pattern in the data.
3. Writing the report	All researchers contributed to writing the report and selecting illustrative quotes.

## Results

### Inter-rater reliability

Our inter-rater reliability was moderate to strong based on McHugh's (2012) interpretation of Cohen’s kappa ( Table 6).

**Table 6.** Calculation of the inter-rater reliability.

Interview Groups	Proportion of interviews double-coded	Average of Kappa	Average of Agreement (%)
Council	1/5 (20%)	0.87	99.79
Remaining Hackney	3/6 (50%)	0.64	99.20
Remaining Lambeth	3/10 (33%)	0.61	98.80

*Note.* Interpretation of Cohen’s kappa is based on McHugh (2012) (value of kappa – level of agreement: 0-.20 – none; .21-.39 – minimal; .40-.59 – weak; .60-.79 – moderate; .80-.90 – strong; above .90 – almost perfect)

### What are the barriers and enablers to walking and cycling for short local journeys in areas where Streetspace measures have been introduced?

**Figure 3.**
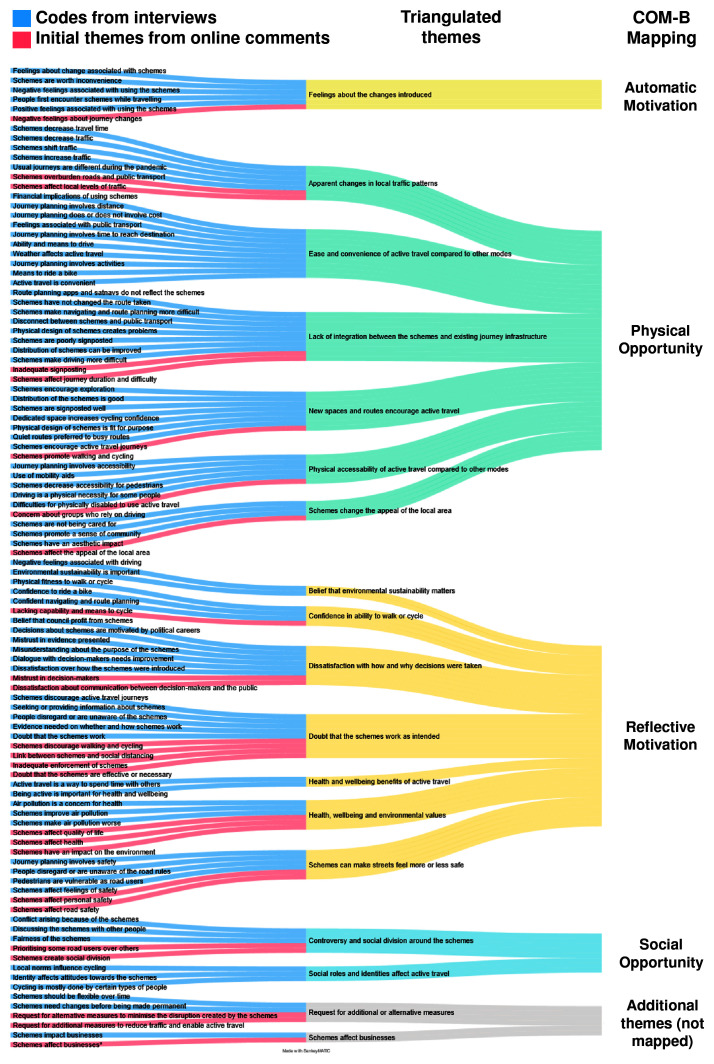
Triangulated themes and mapping to COM-B. *Note.* Codes from the interviews and initial themes from the online comments were generated independently by different researchers. Therefore, the wording for codes and initial themes with similar content may vary. Code names were not unified at that stage since they were merged into triangulated themes in the consecutive step.

We developed 18 themes from the triangulation of the interview transcripts and online comments. Figure 3 shows how each dataset contributed to the final themes. Fourteen themes reflected both datasets and four themes were reflected only in the interviews. Access to the datasets can be found as *Underlying data* ( Hale *et al*., 2023b).

 The themes relate to the overall package of Streetspace measures introduced in the case study boroughs, which could be of four types: temporary cycleways, widened pavements, LTNs, and school streets. The themes are not broken down by type of scheme, as interview participants responded generally about the schemes and had not all encountered all types ( Table 2) and the online comments data did not include widened pavements. During qualitative coding it was noted that interview participants spoke relatively more often about LTNs, school streets and cycleways, whereas widened pavements were less discussed.

Overall, 16 themes were considered to directly address the research question, i.e. described factors which could be barriers or enablers to the target behaviour of walking and cycling for short local journeys instead of driving. These themes were categorised as barriers (5 themes), enablers (3 themes) or mixed (8 themes) and mapped to COM-B ( Figure 3). Table 7 gives example quotes for each theme with the source (interview or online comment, and stakeholder group) and the frequency with which the theme was coded in each dataset. A table mapping the triangulated themes against TDF domains can be found at https://osf.io/gw2rt (Supplementary file 7). In the following sections we describe each theme with reference to the views of ‘participants’; we use the term ‘participants’ to include the interview participants as well as the online commenters.

**Table 7.** Frequencies and illustrative quotes for barrier and enabler themes mapped to COM-B domains.

COM-B domain	Theme ( *n* = 16)	Interviews *n* (max. = 310)	Comments *n* (max. = 239)	Barrier Enabler	Example quotes
Physical Opportunity	1. Apparent changes in local traffic patterns	156	235	Mixed	“It's faster because you don't have to worry about navigating waiting for cars to cross intersections and knowing that there's a ton of traffic” [Interviews: Hackney, schools & families] “It has forced all the traffic onto a handful of main roads. These roads are residential streets. The result has been a living hell for those unfortunate to live on them” [Comments: Hackney, Cycle lane]
	2. Ease and convenience of active travel compared to other modes	192	-	Mixed	“it saves money, I used to spend a lot of money on transport, […] It saves time as well. I think a lot of journeys are actually much quicker.” [Interviews: Hackney, schools & families] “For work, I would drive. As a teacher, I don't teach in the classroom anymore, but before I would always have lots of books and papers and stuff. In terms of weight, it was always easy to drive.” [Interviews: Lambeth, schools & families]
	3. Lack of integration between the schemes and existing journey infrastructure	310	230	Barrier	"if they could link up the black spots would be better where you have to go on the old section of a busy road. If they could eliminate all those, I think more and more people would cycle.” [Interviews: Hackney, community & residents]
	4. New spaces and routes encourage active travel	214	20	Enabler	“now, I walk up to Hyde Park without thinking about it […] For me, the COVID combined with the traffic initiatives have opened up whole new areas of London that I wasn't aware of.” [Interviews: Lambeth, businesses] “We cycled there and we wouldn't have done it if it wasn't for the new cycle routes […] that basically lead you most of the way there.” [Interviews: Hackney, community & residents].
	5. Physical accessibility of active travel compared to other modes	54	93	Barrier	“a lot of these schemes are too narrow for recumbent cyclists, so you need to be on the main road.” [Interviews: Hackney, community & residents] “It’s good to encourage walking but we are forgetting people like me, full working single mother of two […] I would love to walk to school peacefully with my child to school as you are try to impose but this is not possible in certain families.” [Comments: Hackney, School Street]
	6. Schemes change the appeal of the local area	33	59	Mixed	“there seems to be more schemes with people doing community gardening in places where maybe they didn't before.” [Interviews: Lambeth, community & residents] “the LTN planters are hideous looking, real eyes sores.” [Comments: Hackney, LTN]
Social Opportunity	7. Controversy and social division around the schemes	197	129	Barrier	“On both sides, whether it's people that are supportive or people that are against the LTN […] I think a lot of people interpret their experiences of the LTN in the most extreme possible reality.” [Interviews: council]
	8. Social roles and identities affect active travel	18	-	Mixed	“your car is part of your sense of achievement and the LTNs reduce your ability to do that, then you're undoing what I've worked for so long for” [Interviews: council]
Automatic Motivation	9. Feelings about the changes introduced	110	40	Mixed	“[…] if it causes a bit of an irritation to a driver and there are less cyclists killed on the streets and there's less pedestrians being frightened by cars driving too fast, then my irritation is a small price to pay.” (Interviews: Lambeth, businesses) “in an Emergency we are stuck in traffic, this makes it even More STRESSFUL and then with the Virus as well, it is a Nightmare” [Comments: Hackney, cycle lane]
Reflective Motivation	10. Belief that environmental sustainability matters	31	-	Enabler	“the impact that it has on the environment. I think it’s really important, I just don’t know that everybody realizes how important it is to do those things.” [Interviews: Hackney, schools & families]
	11. Confidence in ability to walk or cycle	60	29	Mixed	“I do feel fit enough, but I’m not a confident” [Interviews: Lambeth, schools & families] “I’m an experienced cyclist. […] I don’t need the help in navigating because I know it already. I don’t know how easy it would always be to someone who’s less experienced” [Interviews: Lambeth, community & residents]
	12. Dissatisfaction with how and why decisions were taken	106	154	Barrier	“If they had worked up using feedback, both in Hackney Council and the Mayor’s office as well, then they might have come up with […] schemes which were less disadvantageous” [Interviews: Hackney, community & residents] “I feel the measures have been rushed through” [Comments: Hackney, LTN
	13. Doubt that the schemes work as intended	182	163	Barrier	“They're not designed to support walking because there's increased pollution for a lot of us, and I would think that if you got a smooth traffic flowing that could keep moving, that would be better.” [Interviews, Lambeth, community & residents] “I can see all the cars park here in the morning. It would be good if there's some way of finding out if people have stopped using their cars to come into school because of the school street” [Interviews: Hackney, community & residents]
	14. Health, wellbeing and environmental values	79	239	Mixed	“The improvement to the quality of people's lives, the improvement to the quality of air and the way that some of them also help with COVID restrictions, […] that's all very good. [Interviews, Lambeth, community & residents] “it has had serious consequences on my quality of life: both my partner and I have recently had breathing issues due to the increase in pollution and sleepless nights due to the constant traffic noise.” [Comments: Hackney, cycle lane]
	15. Health and wellbeing benefits of active travel	67	-	Enabler	“I know that it's better for me physically and mentally to walk or to cycle.” [Interviews: Hackney, schools & families] “a friend who I've seen […] We would both cycle, meet at Newington Green, have a cup of coffee outside the park, and cycle around together a bit. That was a great way to spend a bit of time with her.” [Interviews: Lambeth, community & residents]
	16. Schemes can make streets feel more or less safe	195	222	Mixed	“Now some [roads] I won't walk down at night because there's no cars passing by, and I would feel more frightened as a woman if I were on my own.” [Interviews: Hackney, community & residents] “when there are fewer passing cars I feel nervous to walk around the area; I’ve seen several drug deals happening and I felt scared & vulnerable” [Comments: Hackney, cycle lane]

### Barriers

***Physical opportunity***

**Lack of integration between the schemes and existing journey infrastructure.** Participants criticised the integration between new schemes and existing journey infrastructure. They felt that the schemes were not located in the right places, that there were not enough of them, that they lacked continuity or consistency, or needed better integration with the strategic road network and bus routes. Schemes were also considered hard to navigate because of poor signage and not being reflected in route apps and satnavs.

**Physical accessibility of active travel compared to other modes.** Participants described that active travel is not a feasible mode of transport for certain people, including people with disabilities, older people, parents who drop off their children at school, and those who rely on driving for work. Some said the schemes were not designed with those groups in mind and constrained mobility by making car journeys more difficult, even for those who rely on them. Accessibility was also affected by cost and availability of equipment or services to make use of active travel options for all or part of a journey.

***Social Opportunity***

**Controversy and social division around the schemes.** Whether in favour or not, many participants associated schemes with conflict, controversy, polarisation, and social division, while only some thought they were equally fair for all. Certain groups of people were thought to benefit at the expense of others. Cyclists and people living in LTNs were seen as beneficiaries, while car drivers and people unable to use active travel were seen to be disadvantaged. Many mentioned discussing the schemes with others for the purpose of informing or persuading. This theme was prominent in both datasets.

***Reflective motivation***

**Dissatisfaction with how and why decisions were taken.** Participants expressed dissatisfaction with and mistrust in decisions about the schemes. Communication between decision-makers and the public was described as insufficient and frustrating. Many felt the schemes had been rushed through without enough consultation or knowledge of the local area. Some were concerned about motives such as generating profit (e.g. through fines) or pushing personal political agendas.

**Doubt that the schemes work as intended.** Non-council participants expressed doubt that the schemes encourage active travel and support social distancing as intended. This included doubts that they address the right problem, use an appropriate approach, could solve the problem, or that there was no problem to begin with. Consequently, participants sought more evidence about whether and how the schemes work, and many felt they would need more enforcement. In contrast, some council participants endorsed the view that LTNs and school streets were likely to work over time through traffic evaporation (gradual decline in local journeys).

### Enablers

***Physical opportunity***

**New spaces and routes encourage active travel.** Participants described that the schemes and their physical design (including segregated or dedicated space) encourage walking and cycling. Schemes were considered to be well-signposted, located in the right places and adequately interlinked. Some said that the schemes encouraged them to explore their area and find new places or (quieter, preferred) routes.

***Reflective motivation***

**Belief that environmental sustainability matters.** Participants described that sustainability and environmental conservation are important or that this is a decisive factor for their mode of transport. A few expressed guilt about driving a car and the space they take up, which could be used in a more functional and communal way, such as outside café seating. This theme was only coded in the interviews and was one of the least frequently coded.

**Health and wellbeing benefits of active travel.** Participants valued the general positive effects of walking and cycling and the benefits that it has on their physical and mental health and wellbeing. Some described that they are more likely to use active travel if a friend or family member joined them and considered it as an enjoyable way to spend time with others. This theme was only coded in the interviews.

### Mixed themes

***Physical opportunity***

**Apparent changes in local traffic patterns.** Participants had opposing views regarding the effects of the schemes on local traffic patterns. Some felt that the overall amount of traffic and their travel times have decreased. Others perceived the schemes to have caused an increase or shift in traffic onto the main roads, describing gridlock which some implied as challenging the idea of traffic evaporation. People also observed that journey patterns changed due to the pandemic, such as less commuting and an overall reduction in travel.

**Ease and convenience of active travel compared to other modes.** There were mixed views about the ease and convenience of active travel, which could depend on having access to a car, bike (including safe storage), or respective sharing options. On the one hand, walking and cycling were seen to be low-cost, fast, reliable, and flexible. On the other hand, they were considered inconvenient in bad weather, when carrying bulky items, wearing smart clothes, or transporting dependents. For most participants, convenience was considered a more important factor than cost in deciding which mode of travel to take. This theme was only coded in the interviews.

**Schemes change the appeal of the local area.** There were contrasting views about how schemes affected the appeal of the local area. The majority felt they detracted from the neighbourhood, including concerns about appearance, maintenance, noise, and property values. A minority felt that their area had become more pleasant due to reduced noise and a promoted sense of community.

***Social opportunity***

**Social roles and identities affect active travel.** Engaging in travel and support of the schemes was seen to depend on social roles and identities. Some associated cycling with certain stereotypes or groups, especially “fit, young men” (Interviews: council). It was also noted that for some a car is part of their sense of achievement, and that in certain cultures cycling is not seen as aspirational. This theme was only coded in the interviews.

***Automatic motivation***

**Feelings about the changes introduced.** Participants reported mixed feelings about the changes brought about by the schemes, as well as about using the schemes. Whereas some referred to feelings of freedom, hope, and enjoyment, others felt frustrated, stressed, or anxious. Some of whom were part of the latter group, however, appreciated the overall positive effects of the schemes and saw their irritation as a small price to pay overall.

***Reflective motivation***

**Confidence in ability to walk or cycle.** Participants described varying degrees of confidence in their ability to walk or cycle. This was mainly influenced by physical fitness and the behaviour of other road users (e.g. speeding cyclists). They also described mixed levels of confidence in planning a route that involved walking or cycling using the new schemes.

**Health, wellbeing and environmental values.** Perceived effects of the schemes on personal health, wellbeing, and the environment represented barriers for some people and enablers for others. As a barrier, some people described concerns that the schemes increased air and noise pollution to the detriment of their health and quality of life. Some felt “boxed in” (Comments: Hackney, cycle lane). However, others considered the schemes an improvement to the quality of people's lives.

**Schemes can make streets feel more or less safe.** There were mixed views about the effect of the schemes on road and personal safety, which were considered important factors affecting people’s choice of transport. While some described an increased sense of road safety for cyclists, others described that they felt less safe and that they would avoid some roads because of fewer cars passing by. Other safety concerns included worries that emergency vehicles would not be able to access streets or would get stuck in traffic caused by the schemes. Generally, pedestrians were considered as the most vulnerable road users in terms of the physical safety risks posed by others.

### Additional themes

Two themes were identified in the data which did not directly address the research question but were considered relevant to understanding how Streetspace schemes could interact with local active travel behaviour in the long term.

**Schemes affect businesses.** This theme was coded 38 times in the interviews and 88 times in the online comments. The schemes appear to have affected businesses differently, depending on their location and goods sold. Businesses in the hospitality sector were described to have benefitted from additional outside seating space. Others described the schemes as “[a]wful for local business and deliveries” (Comments: Hackney, cycle lane), because of the parking restrictions and increased traffic.

**Request for additional or alternative measures.** This theme was coded 28 times in the interviews and 26 times in the residents’ comments. People gave a range of ideas concerning the future of the schemes: “Some schemes will probably stay because there's a benefit all around. Some schemes will have to be taken out, or some different schemes might have to be introduced. It's an evolving process.” (Interviews: council). Requested improvements for the schemes largely aimed at making the schemes fairer and less disruptive to traffic.

## Discussion

This study investigated barriers and enablers to walking and cycling for short local journeys in two case study London boroughs where Streetspace interventions were introduced during the COVID-19 pandemic. Barriers related to physical opportunity (accessibility of active travel and integration of schemes), social opportunity (controversy), and reflective motivation (dissatisfaction with decision-making process and doubt in the schemes). Enablers related to physical opportunity (new routes and spaces) and reflective motivation (beliefs about sustainability and health). Around half of the themes represented mixed factors, meaning they acted as barriers to walking and cycling for some people and enablers to others. They reflected physical and social opportunity (changes to traffic and appeal of the area, and influence of social roles on active travel) and reflective and automatic motivation (feelings around safety and introduced changes, environmental values and confidence in using active travel). None of the barriers and enablers were mapped to the COM-B domain of capability. Two additional themes which emerged from both datasets described effects of the schemes on businesses and requests for additional or alternative measures. Overall, we found general agreement regarding the emerging themes between the two datasets, with some exceptions which we will discuss below.

Our findings suggest that the schemes were perceived to have some of the intended effects to encourage walking and cycling and local exploration, consistent with previous studies focusing on LTNs ( Aldred & Goodman, 2021; Goodman *et al*., 2020). Despite the intention of LTNs and school streets to support active travel by reducing traffic, this was a mixed theme in our findings: council officers endorsed the expectation that traffic evaporation would occur but perceived changed in traffic were more divided among non-council participants, encouraging some and discouraging others from walking and cycling. In line with previous research, the main motivational enablers to walking and cycling were holding beliefs that environmental sustainability matters, and seeing active travel as beneficial for health and wellbeing ( Bird *et al*., 2018; Daley *et al*., 2007; Verplanken *et al*., 2008). Enablers to active travel were generally more prominent in the interviews compared to the online comments, which could be due to the difference between gathering views through a public online platform versus a more probing interview. It should also be noted that the interviews were conducted some months after sampling online comments when some initial resistance to the schemes might be expected to have decreased.

Consistent with other research, we found that accessibility was a barrier to using the schemes and to active travel in general ( Barton *et al*., 2012; Schreuer *et al*., 2019; Stafford & Baldwin, 2018; Transport for All, 2021). While accessibility is sometimes interpreted in relation to personal physical capability, our data suggested that people saw affordances of the built environment (physical opportunity) as the barrier – and thus also a lever for achieving change. The physical design and temporary nature of the schemes were seen by some to impede accessibility and limit effective integration with existing infrastructure for active travel and public transport. These issues have not received significant attention in previous research although they were anticipated in some of the council interviews. The fairness of the Streetspace schemes for certain groups has triggered substantial controversy, similar to schemes in other regions ( Larrington-Spencer *et al*., 2021; Plimmer, 2021). In line with this, previous research suggests that higher perceived intrusiveness is mirrored in lower perceived fairness ( Huber *et al*., 2020). Motivational barriers, such as doubts about the necessity and effectiveness of the schemes and dissatisfaction with the way decisions have been taken, are likely to have contributed to and been influenced by this controversy. While low perceived effectiveness of an intervention is generally associated with policy opposition ( Huber *et al*., 2020), it is unclear whether perceived effectiveness was formed retrospectively. These motivational themes featured particularly strongly in the online comments sample, which allowed us to include a large number of people’s views, but may also reflect self-selection bias, and the influence of features to see ‘likes’ and share other people’s comments. In line with our sample, previous research suggests that people are more inclined to leave negative online comments than positive ones ( Wu *et al*., 2016).

Half of the themes developed from our data were mixed, reflecting disagreement about whether the changes to the local built environment were beneficial or not. Mixed themes included the effects of the schemes on traffic and air pollution, the appeal of the local area, and on perceived safety, as well as more general influences on active travel that have previously been noted in literature (not specific to the Streetspace schemes) such as confidence in ability to walk or cycle ( Bird *et al*., 2018; Sersli *et al*., 2019), ease and convenience ( Lu *et al*., 2014; Michail *et al*., 2021; Willis *et al*., 2015), and the influence of social roles and identities ( Daley & Rissel, 2011; Engbers & Hendriksen, 2010; Guell *et al*., 2012; Willis *et al*., 2015). Our study adds to existing research suggesting that these more general influences are experienced differently by groups within society and are linked to existing social inequality ( Burns *et al*., 2020; Goodman & Aldred, 2018; Mackett, 2014; Olsen *et al*., 2017; Transport for All, 2021). The Streetspace schemes we studied were not specifically designed to address social roles, identities or inequalities, but our findings point to the relevance of these factors in the uptake of active travel interventions ( Mullen *et al*., 2014; Titheridge *et al*., 2014). A systematic review of infrastructural interventions to promote cycling found that less than 10% of the studies evaluated fairness, advocating for a stronger consideration this factor in future studies ( Mölenberg *et al*., 2019).

Given that schemes were not necessarily distributed similarly across participants’ neighbourhoods, participants may be more or less familiar with individual schemes (see Table 2). Although the majority of our findings related to all four types of schemes (LTNs, school streets, cycleways, and pavement widening), pavement widening was discussed less than other types (and not included in the online comments). We also noted differences in how positively or negatively some types of schemes were viewed. In particular, school streets received the most support out of the four, although people still questioned whether they were fairly distributed across their borough. School streets might have received the most support because people consider them less inconvenient than other schemes, or more acceptable for the purpose of protecting children’s health and safety. LTNs, on the other hand, were particularly divisive as has been reflected in discourses on social media and the news (e.g. Plimmer, 2021; Prosser, 2021). The widening of pavements received little attention, which could reflect that these schemes were clearly implemented to facilitate physical distancing, and that online comments were not specifically sought for them.

As Streetspace schemes have been introduced on a temporary and emergency basis, they are expected to be adaptable and subject to ongoing evaluation and modification. Our study provides two types of insight that are can be considered when modifying, planning, and implementing future active travel interventions. First, our analysis showed that residents were looking for changes to the design or roll-out of the schemes moving forward. This includes providing more information, increasing participation and transparency in the decision-making process, altering the physical design of temporary infrastructure, revising the distribution and connectedness of the schemes, and greater enforcement and maintenance of the schemes. Second, the framework that we used for data analysis (the COM-B model) can be a helpful in identifying additional or alternative ways to address barriers that people encounter whilst using the schemes. To give an illustrative example, the Behaviour Change Wheel (BCW) framework (which is based around the COM-B model) highlights that physical opportunity barriers may be addressed through environmental restructuring, restriction, training, and enablement. Environmental restructuring (e.g. of street features) and restriction (e.g. of vehicles) are already features of the temporary schemes, whereas training (e.g. cycle training) and enablement (e.g. providing social support, material or financial resources) are other intervention types that the BCW indicates as helpful to address physical opportunity. At the time of the study, the case study boroughs offered cycling courses separately to the Streetspace schemes. Further research and deliberation is needed to develop options using the BCW and assess their suitability, using intervention evaluation tools such as the APEASE criteria (acceptability, practicability, effectiveness, affordability, spill-over effects / side effects, equity) ( Michie *et al*., 2014). Systematically applying the BCW in this way could highlight solutions not previously considered by local authorities or residents.

Our data points to the important role that perceived fairness, acceptability, and unintended consequences may have in the overall uptake and success of behavioural interventions. These threads were prominent throughout the comments and interviews we analysed, and were often explicitly or implicitly described as limiting the expected success of the schemes (although a formal evaluation would be needed to confirm whether these expectations played out).Whilst local authorities (including those we studied) have processes in place to prioritise and address these types of concerns, the themes in our data highlight that additional efforts may be needed, particularly when facing urgent pressure to intervene quickly to address health and environmental emergencies. To help achieve this, large bodies of research in public health and sustainability point to the value of participatory approaches based around systems thinking ( Antunes *et al*., 2015; Davies *et al*., 2021; Eker *et al*., 2017; Zimmerman *et al*., 2016). Such approaches bring together stakeholders for open discussion about a problem and aim to integrate their different views into a joint understanding, often through creating a map or model of the system underlying the problem. Behavioural systems mappings a recent extension of these approaches which may be used in conjunction with the BCW to help consider the APEASE criteria and select among different intervention options ( Hale *et al*., 2022a).

Our findings should be interpreted in light of several strengths and limitations of the research. Finding consistent themes between the two independently and inductively coded data sources and using a formal saturation method for the analysis of the online comments raised our confidence that we have identified the main barriers and enablers to local active travel experienced by our participants. While triangulation is considered a way to increase the inclusion of diverse views, one should note the very different nature of the two data sets. In contrast to the interview data, the online comments were often less profound, more polarising, and represented an oppositional attitude towards the schemes more often. This dataset was also subject to self-selection of people who wish to give online feedback. Taken individually, the datasets allow for partly similar and partly divergent conclusions. This is an important insight, considering that online comments are a frequently used method for councils to gather public opinions.

Use of the COM-B model to inform our interview guide meant that we were able to probe a comprehensive set of theoretical constructs which could affect active travel behaviour. However, our interview sample lacked representation from the health and social care sector and from groups traditionally under-represented in research (specifically ethnic minorities and faith groups) while background characteristics were not captured in our sample of online comments. Therefore, it would be important to validate the accuracy and completeness of our results with all stakeholders before applying the findings. While all participants were invited in their role as representative of a stakeholder group, they varied in the extent they drew from personal experience. Additionally, our data did not measure the change in active travel behaviour (e.g. mode shift), nor test causal mechanisms about how behaviour changes. For example, it is unclear whether negative perception of the decision-making process affected participants’ active travel behaviour or whether the perception formed retrospectively.

It is worth noting that in the present study we pooled insights across two case study locations, multiple types of temporary interventions, and different time-points during the pandemic. This has advantages for the comprehensiveness and generalisability of our findings. However, the nature of our participants’ encounters with the schemes (which could be partial or intermixed) means that our findings cannot easily be interpreted at the level of individual types of interventions. Further research is needed to understand how the results generalise to other locations or types of interventions.

## Conclusion

In this study we found that although temporary Streetspace interventions were perceived to have some of the expected benefits for enabling active travel, their long-term success for increasing local walking and cycling could be affected by context-specific and more widely known barriers to active travel. Barriers and enablers we identified were mainly related to opportunities afforded by the physical environment as a result of introducing the schemes, and reflective beliefs and attitudes about the impact of the schemes on the local environment and people’s wellbeing. Attitudes were highly polarised, and this was partly connected to underlying concerns that the schemes could have undesirable consequences for certain groups of people, especially those who are already socially disadvantaged or experience obstacles to active travel. Our study suggests that future local active travel interventions may benefit from strengthening processes to ensure the acceptability and fairness of schemes and avoid unintended consequences, particularly in the face of current and future pressures to address health and environmental emergencies. 

## Data Availability

Open Science Framework: Barriers and enablers to local active travel during COVID-19: A case study of Streetspace interventions in two London. https://doi.org/10.17605/OSF.IO/SCGD2 ( Hale *et al*., 2023b). This project contains the following underlying data: Data → Data Online Comments Sampled from Commonplace (Supplementary File 6).xlsx ( https://osf.io/w9suy) Anonymised interview transcript data are available upon request. To apply for access, contact the CUSSH project Principal Investigator (Prof Michael Davies, m.davies@ucl.ac.uk) and Scientific Manager (Dr Ioanna Tsoulou, i.tsoulou@ucl.ac.uk), with a summary of the intended research for which the data would be used. Access to the data will be granted under the condition that the intended research complies with the British Psychological Society Code of Human Research Ethics, a copy of institutional ethical approval for the intended research is provided, and the request has been reviewed and approved by the UCL Bartlett School of Environment, Energy and Resources (BSEER) Ethics Committee Chair. Open Science Framework: Barriers and enablers to local active travel during COVID-19: A case study of Streetspace interventions in two London. https://doi.org/10.17605/OSF.IO/SCGD2 ( Hale *et al*., 2023b). This project contains the following extended data: Checklists for Qualitative Research and Thematic Analysis → ISSM COREQ Checklist (Supplementary File 1).pdf ( https://osf.io/pa9wv) Checklists for Qualitative Research and Thematic Analysis → 15-Point Checklist of Criteria for Good Thematic Analysis (Supplementary File 2).pdf ( https://osf.io/56pye) Interview guides → Interview guide - Residents (Supplementary File 3).pdf ( https://osf.io/j2y87) Interview guides → Interview guide - Council (Supplementary File 4).pdf ( https://osf.io/mwj28) Participant communication → Handout to participants - Streetspace measures Lambeth, Hackney (Supplementary File 5).pdf ( https://osf.io/tjp5q) Data are available under the terms of the Creative Commons Attribution 4.0 International license (CC-BY 4.0).
