# Supplementary material for: Barriers and enablers to local active travel during COVID-19: A case study of Streetspace interventions in two London boroughs
Source: Wellcome Open Res. 2023 Apr 18;8:177. [Version 1] doi: 10.12688/wellcomeopenres.19164.1 (PMC10468679; doi:10.12688/wellcomeopenres.19164.1)
Supplement: Peer review history

# Peer review: Reviewer response for version 1

**DOI:** 10.21956/wellcomeopenres.21244.r61719

**Published:** 2023-08-30

**Authors:** Annika Aebli

**Affiliations:** 1 University of Lausanne, Lausanne, Switzerland

This study aims to understand the role of temporary ‘Streetspace for London’ schemes in creating barriers or enablers to walking and cycling for short local journeys during the COVID-19 pandemic. Interviews were conducted with a range of stakeholders in Inner London, and online comments on a public web platform were analysed. The two sets of data were analysed thematically; findings were triangulated and categorised using the behavioural science COM-B model.

 General comment: The research is presented with clarity, and is well positioned within current literature on enablers and barriers to active travel. The study design is flawless, the results are well-organised, and the conclusions drawn are adequately supported by the results. Overall, the manuscript is very well written and of good quality.

 One broad issue I would raise for consideration by the authors is:

 A more comprehensive distinction between cycling and walking would be worthwhile since these two modes differ in many ways, such as socio-demographic profile of the user groups. Although some distinctions are discussed in the literature review and the reporting of the findings and subsequent discussion, I think a more thorough distinction between the two modes and groups could be made throughout the article.

 Specific comment:

 The study treats the two boroughs as ‘case studies’. A case study typically requires an in-depth, detailed examination of a particular case. Even though the two ‘cases/contexts’ have been described, I don’t think the authors have conducted an actual case study. ‘Context’ might be a more adequate phrasing.

 Interview materials: what is meant by ‘semantically informing’ the interview guide development?

 Results: Were there differences in the different groups that were interviewed? Some references to the specific groups are made within the results, but I think the results section would benefit from an additional paragraph on grouping according to who was interviewed, e.g. council officers, families, etc. and highlighting the main differences or commonalities between them.

Is the work clearly and accurately presented and does it cite the current literature?

Yes

If applicable, is the statistical analysis and its interpretation appropriate?

Not applicable

Are all the source data underlying the results available to ensure full reproducibility?

Yes

Is the study design appropriate and is the work technically sound?

Yes

Are the conclusions drawn adequately supported by the results?

Yes

Are sufficient details of methods and analysis provided to allow replication by others?

Yes

Reviewer Expertise:

Persuasive technology, gamification, motivation, behaviour change, physical activity.

I confirm that I have read this submission and believe that I have an appropriate level of expertise to confirm that it is of an acceptable scientific standard.

# Peer review: Reviewer response for version 1

**DOI:** 10.21956/wellcomeopenres.21244.r63765

**Published:** 2023-08-16

**Authors:** Celia Kingsbury

**Affiliations:** 1 university of Montreal, Montréal, QC, Canada

**Specific comments**

**Introduction**

- It would be relevant to add a more detailed description of the studied interventions (eg. how were the neighbourhoods receiving the intervention chosen, how many were they?)
- What does "temporary" means in "temporary active travel schemes"? What was the duration of the intervention? Were schools and shops closed during the intervention? During the data collection?
- "The COM-B model has been applied to understanding health and transport behaviour during the pandemic". What are the results?
- When exposing the resume of the literature on the barriers and enablers to active travel, levels of the socioecological model should be precise (eg., at the individual level, at the social level, built environment, etc.). Also, I suggest distinguishing cycling and walking, since they are two different active transport behavior and are associated with different determinants ( https://doi.org/10.1016/j.tra.2018.08.023)
- What is defined as "short journey"? Is it the same for cycling and walking?
- Were parking places removed in some of the interventions?
- In the research question, I suggest adding "perceived" barriers and enablers.

**Methods**

- It would be relevant to add more sociodemographic information about the two boroughs, especially because gender for example are associated with lower cycling rates ( https://doi.org/10.1016/j.jth.2020.100871)
- How is poverty measured?
- Acceptability of the project?
- How much time between the interviews and the implementation of the interventions?
- Do you have the profile of participants who completed the online survey? This would be relevant, especially in if you are interested in sociodemographic disparities
- Do we know if interviewed participants also participated in online comments?
- Is it possible to associated specific elements of the COM-B model  with a specific intervention and a specific group?

**Analyses**

- Why analyzing schemes separately, since they generate different behaviors and experience? Please further detail this choice

**Results**

- Why not grouping results accordingly to who was interviewed? Eg. council officers, families, etc. This would help understand how acceptability is distributed among groups

**Discussion**

- "Around half of the themes represented mixed factors, meaning they acted as barriers to walking and cycling for some people and enablers to others". When saying "for some people...to others", who?
- How do you translate results on the long term?
- Please give more indication about how public policies could use of these results

Is the work clearly and accurately presented and does it cite the current literature?

Yes

If applicable, is the statistical analysis and its interpretation appropriate?

Partly

Are all the source data underlying the results available to ensure full reproducibility?

Partly

Is the study design appropriate and is the work technically sound?

Partly

Are the conclusions drawn adequately supported by the results?

Yes

Are sufficient details of methods and analysis provided to allow replication by others?

Yes

Reviewer Expertise:

Public health, health promotion, active travel, built environment, health inequalities, physical activity, behavior change

I confirm that I have read this submission and believe that I have an appropriate level of expertise to confirm that it is of an acceptable scientific standard, however I have significant reservations, as outlined above.

## References

[1] : Cycling or walking? Determinants of mode choice in the Netherlands. *Transportation Research Part A: Policy and Practice* .2019;123: 10.1016/j.tra.2018.08.023 7-23 10.1016/j.tra.2018.08.023

[2] : Beyond the bicycle: Seeing the context of the gender gap in cycling. *Journal of Transport & Health* .2020;18: 10.1016/j.jth.2020.100871 10.1016/j.jth.2020.100871

# Peer review: Reviewer response for version 1

**DOI:** 10.21956/wellcomeopenres.21244.r56417

**Published:** 2023-05-23

**Authors:** Caroline Bartle

**Affiliations:** 1 Centre for Transport and Society, University of the West of England, Bristol, England, UK

**Summary**

 The article reports on a qualitative study of the role of temporary ‘Streetspace for London’ schemes in creating enablers and barriers to walking and cycling during the COVID-19 pandemic.  Interviews were conducted with a range of stakeholders in the London boroughs of Lambeth and Hackney, and online comments on a public web platform were analysed (the platform was being used by the two local councils to collect public feedback on the schemes). The two sets of data were analysed thematically; findings were triangulated and categorised using the behavioural science COM-B model.    

**General comments**

**Is the work clearly and accurately presented and does it cite the current literature?**

 The research is presented with clarity, and is well positioned within current literature on enablers and barriers to active travel. The description of methods is very clear, and well supported by information provided in supplementary files. The analysis adds to current understanding by raising, in particular, the issue of perceived fairness when interventions are introduced rapidly amid a crisis.

 Two broad issues I would raise for consideration by the authors are:

1. Although ‘active travel’ has become a familiar combined term, cycling and walking differ in many ways, including differences in socio-demographic profile and differences in the way the two modes are perceived (cycling tending to attract more polarised opinions among the public and in the media). In the article some distinctions are made between the two modes in the literature review and in the reporting of the findings and subsequent discussion, but I felt this could have been done more comprehensively through the article.   
  For example at the end of paragraph 6 of the Introduction it is stated that *“demographic groups who are disproportionately negatively affected by the pandemic coincide with groups who experience more (or more pronounced) financial and social barriers to active travel, including women, people with disabilities, older people, people of lower socio-economic status, and people from ethnic minorities.” *Is it the case that all of these groups are more likely to experience barriers to both cycling *and* walking (women, for example?).
  Furthermore, there can be tensions between the two modes when it comes to the allocation of public space (as alluded to in paragraph 5 of the introduction: *“Opponents of the active neighbourhoods were often not motorists, as one might assume, but included people who walk exclusively for their local journeys.”*). An *enabler* for cycling might become a *barrier* to walking, rather than both forming a coherent whole as ‘active travel’.
*  *
2. Schemes such as Streetspace were set up principally to allow greater physical distancing on streets at times in the pandemic when people were urged to stay in their locality and to limit excursions outside the home to exercise or essential tasks such as shopping. Cycling and walking experienced a sharp rise in popularity, not just as a means of (active) travel, but also for leisure and wellbeing. 
  In the article it is not entirely clear how far the schemes were creating enablers and barriers to walking and cycling for utility travel (i.e. to get from A to B), or to walking and cycling for leisure and wellbeing in the specific context of the pandemic. This could affect the generalisability of the results – something that could perhaps be discussed more in the article.  

**Specific comments**

**Is the study design appropriate and is the work technically sound?**

 The research design is clear and appropriate.

 In the section on the Case Study Boroughs, it is noted that “ *Given the similarities and differences between the boroughs, the findings were expected to include a wide range of views which could be meaningfully integrated and aid generalisability.”* It would be useful to say a little more about the type of generalisability aimed for in the study, and how the use of two study locations aided generalisability.

 In Table 1, two of the characteristics could be clarified:  

- ‘car ownership rate’ – is this per household?
- ‘Daily journeys made by Walking, Cycling and Public Transport’ – what does the percentage denote? E.g. is it all journeys over a certain distance?

**Are sufficient details of methods and analysis provided to allow replication by others?**

- A commendably thorough explanation is provided of the methods and analysis process (e.g. Tables 3 and 4). A few specific comments are below.   
- Interview participants – please define ‘APA style’.
- ‘Businesses’ - what type of people within what type of businesses?
- What type of community groups were approached?

Table 2 – typo – ‘officersa’

 Interview materials – what is meant by ‘semantically inform’?

 Online comments – how was the sampling period (6 months up to Feb 2021) decided upon?

 Why were there *‘not online comment data available for widened pavements’* (please note typo), despite pavement-widening being potentially important for encouraging walking and facilitating physical distancing? This also seems relevant to the finding that *“* *Generally, pedestrians were considered as the most vulnerable road users in terms of the physical safety risks posed by others.” * 

 Epistemology

 The authors could specify that the results are people’s *perceptions* of enablers and barriers, as this has implications for how we interpret the findings, and what policy-makers should do to improve interventions. Are there times, for example, when perceptions should be addressed separately from what might be called ‘objective’ barriers?

 It would be useful to explain briefly how the analysis is grounded in the essentialist/realist paradigm and how this connects to the use of explicit rather than interpretive coding.

**Are the source data underlying the results available to ensure full reproducibility?**

 Data in the form of online comments are provided in Supplementary file 6; however the format does make it quite difficult to read the comments in full.

**Are the conclusion drawn adequately supported by the results?**

 COM-B – I wondered why no codes were mapped on to Capability, specifically *Confidence in ability to walk or cycle. *The following is classed as reflective motivation, but could it not also be described as ‘capability’?

*“Participants described varying degrees of confidence in their ability to walk or cycle. This was mainly influenced by physical fitness and the behaviour of other road users (e.g. speeding cyclists). They also described mixed levels of confidence in planning a route that involved walking or cycling using the new schemes*.” 

 Table 6  – please could the authors explain how the ‘average of Agreement’ (%) relates to the Kappa rating?

 Figure 3 is very difficult to read (very small). I’d suggest dividing it into two slides in the ppt. version.

 As raised in my earlier comments, it would be interesting to know whether some of the results concerned cycling more than walking. For example, were the responses biased towards controversy around space given to cycling rather than to walking? In the ‘Social opportunity’ category, were social roles ascribed more to ‘cyclists’ than pedestrians?

**Discussion section**

 Paragraph 2 – typo: ‘perceived changed in traffic’.

 Paragraph 6 typo - “Our study provides two types of insight that are can be considered”.

 Paragraph 5 – It would be interesting to know the authors’ thoughts here on whether the overall results might have been influenced by the paucity of comments on the widening of pavements. Had comments on this been sought, might this perhaps have changed the balance of argument for and against the ‘active travel’ measures as a whole, particularly with regard to issues such as accessibility and fairness, as wider pavements might be seen as improving accessibility for some?  

**Supplementary files**

 Supplementary file 5 – the photos are very useful to help the reader visualise some of the the infrastructure changes. It would be good to alert the reader to them, in the main text, early in the article. Similarly, one of the findings is that schemes change the appeal of the local area (‘Concerns about appearance’). A reference here to the photos would also be useful to the reader.  

 Note that Supplementary file 7 is not listed at the end of the article with the other supplementary files.

Is the work clearly and accurately presented and does it cite the current literature?

Yes

If applicable, is the statistical analysis and its interpretation appropriate?

Not applicable

Are all the source data underlying the results available to ensure full reproducibility?

Yes

Is the study design appropriate and is the work technically sound?

Yes

Are the conclusions drawn adequately supported by the results?

Partly

Are sufficient details of methods and analysis provided to allow replication by others?

Yes

Reviewer Expertise:

Sustainable transport and travel behaviour

I confirm that I have read this submission and believe that I have an appropriate level of expertise to confirm that it is of an acceptable scientific standard, however I have significant reservations, as outlined above.
